# Improvement of simultaneous genome editing of homoeologous loci in polyploid wheat using CRISPR/Cas9 applying tRNA processing system

**DOI:** 10.5511/plantbiotechnology.25.0214b

**Published:** 2025-06-25

**Authors:** Shoya Komura, Mitsuko Kishi-Kaboshi, Fumitaka Abe, Yoshihiro Inoue, Kentaro Yoshida

**Affiliations:** 1 Graduate School of Agriculture, Kyoto University; 2 Kazusa DNA Research Institute; 3 Institute of Crop Science, National Agriculture and Food Research Organization

**Keywords:** CRISPR/Cas9, genome editing, polyploid, tRNA processing system, wheat

## Abstract

Wheat (*Triticum aestivum* L.) consists of three genomes, and notable mutant phenotypes can be observed when all homoeologs are knocked out due to functional redundancy among the homoeologous gene copies. Therefore, high editing efficiency is required to rapidly obtain loss-of-function mutants in wheat. The endogenous tRNA processing system of CRISPR/Cas9 genome editing enables the expression of multiple single-guide RNA (sgRNAs) under the control of a single promoter, facilitating simultaneous multiple genome editing in an organism. Here, we evaluated the genome editing efficiency of multiple sgRNA expressions with the tRNA processing system. At first, using sgRNA of quantitative trait locus for seed dormancy 1, polycistronic tRNA–sgRNA vectors were introduced into immature embryos, and genome editing efficiency was evaluated in the transformed T_1_ plants. In the use of three sgRNA modules, there was no difference in the efficiency of genome editing among the positions of the sgRNAs. We subsequently tested simultaneous genome editing of multiple homoeologous loci. Simultaneous expression of six sgRNAs per gene to target all homoeologous loci increased the editing efficiency of all homoeologous loci up to 100%. Our study indicates that the tRNA processing system is highly effective at simultaneous genome editing of homoeologous loci of wheat.

Wheat is an essential crop. Climate change has prompted the development of new stress-tolerant cultivars. As genome sequences of wheat cultivars accumulate, expressed genes are being revealed by transcriptome sequencing (Walkowiak et al. 2020; White et al. 2024), but their functions remain largely unknown. Allohexaploid wheat (*Triticum aestivum* L.) has triplet homoeologous loci in the A, B, and D genomes. About 70% of genes show balanced expression among the three homoeologs (Ramírez-González et al. 2018). Since gene function is conserved among the homoeologs, single and double knockout mutants usually exhibit the same phenotype as wild-type plants (Wang et al. 2014), as functional redundancy allows the homoeologs to complement the function of knockouts. To genetically elucidate the function of wheat genes, knockout of all homoeologs is therefore required.

The use of “clustered regularly interspaced short palindromic repeats” (CRISPR)/CRISPR-associated protein 9 (Cas9) genome editing in plants has accelerated the elucidation of gene function and crop improvement (Zhang et al. 2020) through the precise mutation of targeted genomic regions. Genome editing of wheat with CRISPR/Cas9 has been used to elucidate gene function and to improve agronomically important traits (Yigider et al. 2023). For example, four grain-quality-related genes—*TaPinb*, *TaWaxy*, *TaPPO*, and *TaPSY*—were independently genome-edited with single sgRNA per gene (Zhang et al. 2021). In the T_0_ generation, the rate of editing of *TaPinb* and *TaPPO* was 0%. Those of *TaWaxy* and *TaPSY* were 33.3% and 6.3%, but the edited alleles were heterozygous. Thus, two more generations were needed to obtain homozygous edited alleles of all homoeologs of these genes. In the genome editing of *TaQsd1* with a single sgRNA (Abe et al. 2019), homozygous edited alleles of each homoeolog are not obtained in the T_0_ generation, and more generations are necessary to obtain biallelic edited alleles of all homoeologs. However, simultaneous genome editing of all homoeologs in wheat is still challenging and requires multiple generations to obtain edited alleles of all homoeologs. To increase genome editing efficiency in wheat, improvements in *Cas9* coding sequences or transformation steps were achieved (Kishi-Kaboshi et al. 2023; Lawrenson et al. 2024). However, further approaches are needed to generate loss-of-function mutants quickly.

The development of an endogenous tRNA processing system of CRISPR/Cas9 allows editing of multiple genes (Xie et al. 2015). A polycistronic tRNA–sgRNA array is constructed by linking multiple sgRNAs flanked by tRNAs (Supplementary Figure S1). It is expressed under the control of a single RNA polymerase III promoter. The primary transcript is cleaved by endogenous RNase P and RNase Z targeting tRNA, generating multiple processed sgRNAs. This system has been used for simultaneous genome editing of *TaGW2*, *TaLpx-1*, and *TaMLO* in hexaploid wheat (Wang et al. 2018). A single sgRNA for each gene was designed and integrated into the array, and regenerated plants with knockout mutations of *TaGW2* or *TaMLO* were obtained. However, only 1 of 39 transgenic T_0_ plants (2.56%) had edited alleles in the homozygous state among all homoeologs of *TaGW2*.

Here, we applied the endogenous tRNA processing system of CRISPR/Cas9 genome editing to hexaploid and tetraploid wheats via *Agrobacterium*-mediated transformation to verify sgRNA excision by tRNA processing and differences in genome editing efficiency among sgRNA positions within the tRNA–sgRNA arrays. To improve the efficiency of acquisition of biallelic or homozygous edited alleles of all homoeologs in one generation, we designed multiple sgRNAs targeting each gene, integrated them into the arrays, and evaluated genome editing efficiency in regenerated transformed plants.

We used a modular cloning tool kit based on the Golden Gate cloning method to construct vectors (Engler et al. 2014; Hahn et al. 2020). We used sgRNA of *Quantitative trait locus for seed dormancy 1* (*Qsd1*) (Abe et al. 2019) and designed sgRNAs of *LUX/PHYTOCLOCK 1* (*PCL1*) and *B-Box 19* (*BBX19*) in DeepHF sgRNA prediction software (Wang et al. 2019). The on-target and potential off-target of all designed sgRNA were confirmed using GrainGenes BLAST service (Yao et al. 2022). Target sequences were integrated into tRNA–sgRNA backbone vectors according to Hahn et al. (2020). All sgRNAs are listed in Supplementary Table S1. The tRNA–sgRNA arrays were assembled downstream of the TaU3 promoter (TaU3pro). *T. aestivum WUS homeobox-containing 5* (*TaWOX5*) was introduced to increase transformation efficiency and callus regeneration frequency (Wang et al. 2022). *Hygromycin phosphotransferase II* (*Hpt*) plus *turbo GFP* (*tGFP*) and *SpCas9* were introduced as selection markers. The design and construction of the vectors are illustrated in Supplementary Figure S2. All vectors are listed in Supplementary Table S2.

Hexaploid wheat cultivar ‘Fielder’ and tetraploid wheat cultivar ‘Kronos’ were *Agrobacterium*-transformed according to Abe et al. (2020). The embryos were infected with *Agrobacterium* strain EHA101 with an OD_600_ of 0.5 and incubated in co-cultivation medium for 2 days. The embryo axes were removed and the scutella were transferred to resting medium and incubated at 24°C in the dark for 7 to 10 days for differentiation. Calluses were transferred onto the first selection medium containing 15 mg l^−1^ hygromycin and incubated at 24°C in the dark for 2 weeks. Calluses were then moved to the second selection medium containing 30 mg l^−1^ hygromycin, and resistant calluses were transferred onto regeneration medium and regenerated at 23°C under 16-h light. The regenerated plants were moved onto the rooting medium until the roots were fully elongated and then planted in soil. All media are described in Abe et al. (2020).

To confirm genome editing, we extracted genomic DNA from leaf tips of T_0_ plants with a DNeasy Plant Mini Kit (Qiagen, Hilden, Germany). The target loci were amplified using primer sets listed in Supplementary Table S3. Plants were classified as transformed or untransformed on the basis of the amplification of *Hpt*. To confirm genome editing of *Qsd1*, we performed PCR-RFLP analysis (Abe et al. 2019). Genome editing of *PCL1* and *BBX19* in the transformants were detected by Sanger sequencing using subgenome-specific primers.

To examine differences in genome editing efficiency among sgRNA positions in the polycistronic tRNA–sgRNA array, we performed genome editing of sgRNAs among which the position of *Qsd1* sgRNA and the number of sgRNA modules differed (Figure 1A, B). The three modules were transformed into ‘Fielder’ at efficiencies of 16.7% to 20.0% (Table 1), indicating that the number of modules (1–3) did not affect the efficiency of transformation of hexaploid wheat. When a single sgRNA module was transformed, 88.9% of transformants showed genome editing of *Qsd1*. When double and triple sgRNA modules were transformed, the rate increased to 100% (Table 1, Figure 1C). The result of Sanger sequencing confirmed mutations at the *Qsd1* target sites of T_1_ plants obtained from two of each T_0_ transformant of the single, double and triple sgRNA modules (Supplementary Figure S3). These results indicate that sgRNA excision by tRNA processing worked, and differences in sgRNA positions in the array are independent of the genome editing efficiency.

**Figure 1:**
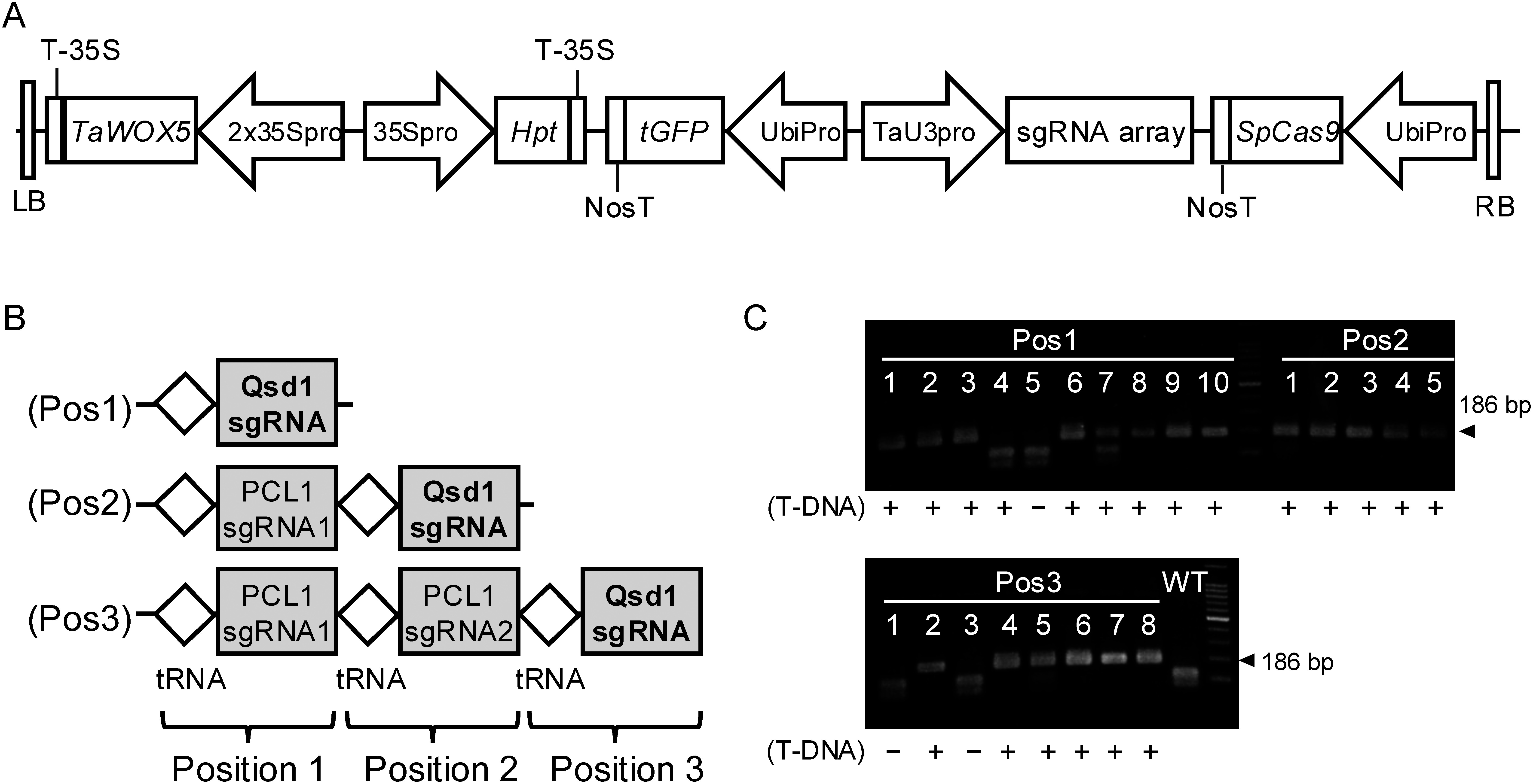
Figure 1. Evaluation of endogenous tRNA processing system of CRISPR/Cas9 genome editing using sgRNA of *Qsd1*. (A) Schematic diagram of transformation vectors. (B) Structure of the three sgRNA modules. 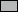 sgRNA, ◇ tRNA. The position of *Qsd1* sgRNA varies by module. (C) PCR-RFLP analysis for confirming the *Qsd1* genome editing in the T_0_ plant. In wild-type allele, the 186 bp PCR product was digested with PstI. On the other hand, amplicons from genome-edited alleles were not digested. + and − indicate the presence or absence of T-DNA determined by the amplification of *Hpt* fragment.

**Table 1:** Table 1. Efficiency of transformation and genome editing at different *Qsd1* sgRNA positions.

Vector construction	Number of immature embryos^a^	Number of PCR-positive T_0_ plants^b^	Transformation efficiency	Number of mutants/transformants (%)	Number of mutants/inoculated embryos (%)
Pos 1	51	9	17.6%	8/9 (88.9%)	8/51 (15.7%)
Pos 2	30	5	16.7%	5/5 (100%)	5/30 (16.7%)
Pos 3	30	6	20.0%	6/6 (100%)	6/30 (20.0%)

^a^ Number of immature embryos used for transformation. ^b^ Number of T_0_ plants showing PCR amplification of *Hpt*.

To achieve simultaneous multiple genome editing of all homoeologs in one generation, we used six sgRNAs per gene (Figure 2A). The T-DNA vector had two polycistronic tRNA–sgRNA arrays, each of which contained three sgRNA modules (Figure 2B). sgRNAs that target each of *PCL1* and *BBX19* are located in the regions conserved between homoeologs except for sgRNA1 of *PCL1* and sgRNA2 of *BBX19* (Supplementary Table S1). We tested genome editing of ‘Fielder’ and ‘Kronos’. The efficiency of *Agrobacterium*-mediated transformation ranged from 3.0–12.6% (Table 2). The mutations at the target sites of T_0_ transformants were confirmed using Sanger sequencing. Mutations of at least one homoeolog of *PCL1* and *BBX19* were found in 81.8% and 100% of the ‘Fielder’ transformants, respectively. In the transformants of ‘Kronos’, editing efficiencies of at least one homoeolog reached 100% in both targets.

**Figure 2:**
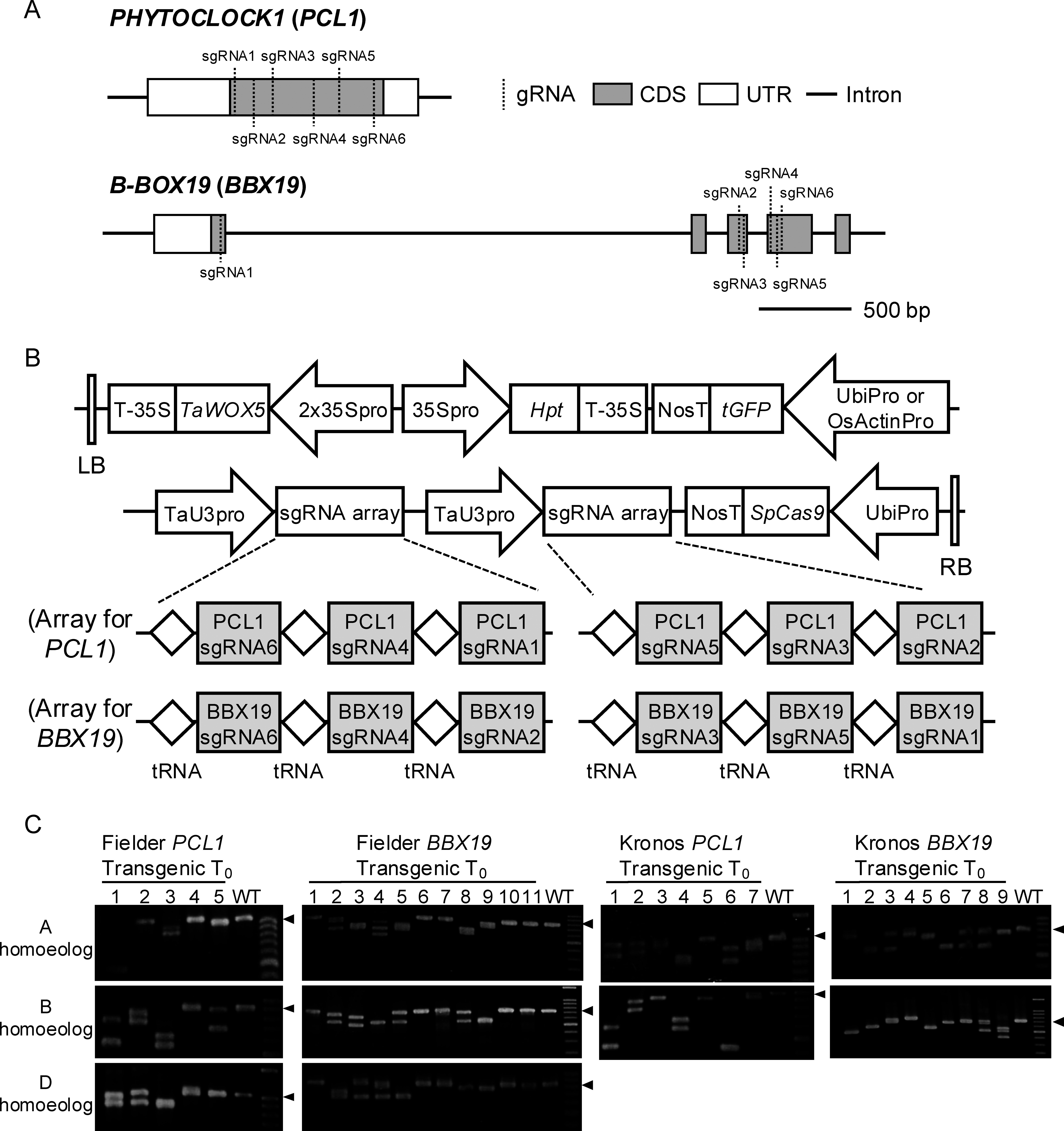
Figure 2. Design of polycistronic tRNA–sgRNA targeting all homoeologous loci. (A) Schematic representation of *PCL1* and *BBX19* sgRNA design. (B) Structure of multiplex genome editing vectors targeting *PCL1* and *BBX19*. The six arrayed sgRNAs were expressed under the control of the wheat U3 promoter. (C) PCR amplification of *PCL1* and *BBX19* in transgenic T_0_ plants of ‘Fielder’ and ‘Kronos’. Black arrowheads indicate PCR products obtained from wild-type ‘Fielder’ or ‘Kronos’ for each target gene. The number on each lane indicates the transgenic ID in Supplementary Tables S7 and S8.

**Table 2:** Table 2. Summary of simultaneous genome editing of homoeologous loci of *PCL1* and *BBX19*.

Genotype	Target gene	No. of immature embryos^a^	No. T_0_ plants^b^	Transformation efficiency	No. of edited plants	
					At least one homoeolog	All homoeologs
Fielder	*PCL1*	100	5	5.0%	5 (100%)	5 (100%)
Fielder	*BBX19*	87	11	12.6%	9 (81.8%)	6 (54.5%)
Kronos	*PCL1*	233	7	3.0%	7 (100%)	7 (100%)
Kronos	*BBX19*	153	9	5.9%	9 (100%)	7 (77.8%)

^a^ Number of immature embryos used for transformation. ^b^ Number of T_0_ plants showing PCR amplification of *Hpt*.

Furthermore, in ‘Fielder’, 100% and 54.5% of the transformants were edited in all homoeologs of *PCL1* and *BBX19* were detected, respectively. In ‘Kronos’, 100% and 77.8% of the transformants were edited in all homoeologs of *PCL1* and *BBX19*, respectively (Table 2). Genome editing efficiencies in each gRNA ranged from 30–100% (Supplementary Table S4). The most frequently detected zygosity in the T_0_ transformants was biallelic of two edited alleles, followed by homozygous of single edited allele and homozygous of non-edited allele (Table 3). The total proportion of knocked out genotypes, including homozygous, biallelic, and chimeric types, was 83.7%. These results indicate that the polycistronic tRNA–sgRNA array system can simultaneously edit the multiple homoeologous loci in one generation with high efficiency.

**Table 3:** Table 3. Summary of zygosity of transgenic T_0_ plants at each homoeologous loci of *PCL1* and *BBX19*.

Genotype	Target gene	No. of T_0_ plants	Homoeologous loci	Number of T_0_ plants				
				Homo^a^	Hetero^b^	Biallelic^c^	Chimera^d^	WT^e^
Fielder	*PCL1*	5	A	1 (20%)	0	4 (80%)	0	0
			B	0	0	5 (100%)	0	0
			D	0	1 (20%)	3 (60%)	1 (20%)	0
	*BBX19*	11	A	2 (18.2%)	0	5 (45.5%)	2 (18.2%)	2 (18.2%)
			B	2 (18.2%)	1 (9.1%)	3 (27.3%)	2 (18.2%)	3 (27.3%)
			D	3 (27.3%)	0	3 (27.3%)	0	5 (45.5%)
Kronos	*PCL1*	7	A	0	0	7 (100%)	0	0
			B	2 (28.6%)	0	5 (71.4%)	0	0
	*BBX19*	9	A	2 (22.2%)	1 (11.1%)	5 (55.6%)	1 (11.1%)	0
			B	4 (44.4%)	0	4 (44.4%)	1 (11.1%)	0
Total		32 (80 target loci)		16 (20.0%)	3 (3.8%)	44 (55.0%)	7 (8.8%)	10 (12.5%)

^a^ Homozygous of single edited allele. ^b^ Edited allele and wild-type allele. ^c^ Two edited alleles with different sequences. ^d^ More than three types of sequences. ^e^ Homozygous of the wild-type allele.

The use of multiple sgRNAs per gene increases the probability of gene knockout. We designed six sgRNAs per gene, enabling the simultaneous editing of homoeologs in both allotetraploid and allohexaploid wheats in one generation. With a single sgRNA, the frequency of simultaneous genome editing of all homoeologs is low (Wang et al. 2018; Zhang et al. 2021). To develop plants with knockouts of all homoeologs, it is necessary to cross mutants with knockouts of different homoeologs or to increase the number of trials for *Agrobacterium*-mediated transformation. In addition, cleavage efficiency is highly dependent on sgRNAs (Kamiya et al. 2020; Milner et al. 2020). It generally takes 2.5 to 3 months to obtain regenerated wheat transformants (Cheng et al. 1997). The use of single sgRNAs runs the risks of being found to be the failure of genome editing after obtaining transformants. On the other hand, our approach knocked out all homoeologs at a rate of 54.5% to 100% in allohexaploid ‘Fielder’ and allotetraploid ‘Kronos’ in one generation, accelerating the development of mutants with knockout of all homoeologs and thus the elucidation of gene function.

Another possible factor for the high efficiency of genome editing is the use of *TaWOX5*: calluses expressing *TaWOX5* could be regenerated with high frequency and preferentially selected (Wang et al. 2022). If T-DNA is inserted into euchromatic regions of a chromosome, not only *TaWOX5* but also *SpCas9* and sgRNA are expected to be highly expressed. If there is a bias that the calluses expressing *TaWOX5* are more vulnerable to genome editing than the non-expressing calluses due to such a factor above, the frequency of genome editing of regenerated wheat could be increased. Moreover, overexpression of *TaWOX5* significantly increased leaf width (Supplementary Table S5). These morphological changes could be useful as a selective marker for transformants. The expression of multiple sgRNAs potentially increases the risk of off-target editing. In fact, mutations were detected in several off-target candidates (Supplementary Table S6). Therefore, more stringent selection steps will be needed to design sgRNA.

The use of six sgRNAs per gene generated longer deletions with high frequency (Figure 2C). These deletions can be detected by 2% agarose gel electrophoresis, whereas genome editing with a single sgRNA usually generates small deletions (Liu et al. 2020; Nekrasov et al. 2013). In the genome editing of *PCL1* and *BBX19*, the edited alleles had different sizes of deletions ranging from 1 bp to 576 bp (Supplementary Tables S7, S8). This observation suggests that the design of multiple sgRNAs for a single gene could generate a wide variety of edited alleles, including those missing specific domains, the terminal portion of the coding region, or most of the coding region. The variety of mutant alleles could be useful for evaluating the functions of protein domains.

## Abbreviations

35Spro: CaMV 35S promoter
BBX19: B-Box 19
CaMV: cauliflower mosaic virus
Cas9: CRISPR-associated protein 9
CRISPR: clustered regularly interspaced short palindromic repeats
Hpt: hygromycin phosphotransferase II
NosT: nopaline synthase terminator
OsActinPro: *Oryza sativa* actin promoter
PCL1: LUX/PHYTOCLOCK 1
Qsd1: quantitative trait locus for seed dormancy 1
sgRNA: single-guide RNA
SpCas9: *Streptococcus pyogenes* Cas9
T-35S: CaMV35S terminator
TaGW2: *Triticum aestivum* grain weight 2
TaLpx-1: *T. aestivum* lipoxygenase 1
TaMLO: *T. aestivum* mildew resistance locus O
TaU3pro: TaU3 promoter
tRNA: transfer RNA
UbiPro: ubiquitin promoter from *Zea mays*
WOX5: WUS homeobox-containing 5

## Description of supplementary files

Supplementary Table S1. sgRNAs used in this study.

Supplementary Table S2. Vectors used in this study.

Supplementary Table S3. Primers used for detection of genome editing.

Supplementary Table S4. Estimation of genome editing efficiency for each sgRNA.

Supplementary Table S5. Comparison of the morphology of plants with and without T-DNA in ‘Fielder’ and ‘Kronos’.

Supplementary Table S6. Potential off-target of designed sgRNAs in this study.

Supplementary Table S7. Summary of genome-edited sequences in *PCL1*.

Supplementary Table S8. Summary of genome-edited sequences in *BBX19*.

Supplementary Figure S1. Overview of the endogenous tRNA processing system of CRISPR/Cas9 genome editing.

Supplementary Figure S2. Schematic representation of vector assembly based on the Golden Gate cloning method.

Supplementary Figure S3. Mutations detected in T_1_ plants derived from selected T_0_ plants transformed with the three sgRNA modules (pos1–3).
