# Supplementary material for: Improvement of simultaneous genome editing of homoeologous loci in polyploid wheat using CRISPR/Cas9 applying tRNA processing system
Source: Plant Biotechnol (Tokyo). 2025 Jun 25;42(2):167–72. doi: 10.5511/plantbiotechnology.25.0214b (PMC12235420; doi:10.5511/plantbiotechnology.25.0214b)
Supplement: Supplementary Data [file plantbiotechnology-42-2-25.0214b-s001.pdf]

Supplementary Table S1. sgRNAs used in this study.

Target	Position in gene	Position in gRNA array	Sequence (5′ to 3′)	Strand	Target homeoloci in 'Fielder'	Target homeoloci in 'Kronos'	DeepHF	Origin
Qsd1			ACGGATCCACCTCCCTGCAG	+	ABD	AB		Abe et al. 2019
PCL1	1	Array1-3	ACGTCGAGAAGAGTCCGCCC	–	A	AB	0.748	This study
	2	Array2-3	GCTGCACAAGCGGTTCGTGG	+	ABD	AB	0.672	This study
	3	Array2-2	CATCATGCAGCTGATGAACG	+	ABD	AB	0.655	This study
	4	Array1-2	GATGCAGGGCCTCTCCAACG	+	ABD	AB	0.746	This study
	5	Array2-1	GGAGGCGAAGATGTGGTCGG	–	ABD	AB	0.708	This study
	6	Array1-1	TACTACCAGCAGCAGCACAG	+	ABD	AB	0.705	This study
BBX19	1	Array2-3	GGTGGCGGTGCTCTTCTGCG	+	ABD	AB	0.697	This study
	2	Array1-3	GAACAGCCATATCACAACTC	–	AB	AB	0.664	This study
	3	Array2-1	GTTGGTGGGAAACGAACCCA	+	ABD	AB	0.742	This study
	4	Array1-2	AGTTTCCAGGAGATAAACCA	+	ABD	AB	0.729	This study
	5	Array2-2	GCTACATCGTCCATATGCCC	–	ABD	AB	0.713	This study
	6	Array1-1	AGATGGAGTCTGAAAACCCA	+	ABD	AB	0.724	This study

Supplementary Table S2. Vectors used in this study.

Name	Description	Reference	Available
pFH113	tRNA-gRNA backbone, Position 1	Hahn et al. 2020	https://www.addgene.org/128210/
pAK002	tRNA-gRNA backbone, Position 2	Hahn et al. 2020	https://www.addgene.org/128215/
pAK003	tRNA-gRNA backbone, Position 3	Hahn et al. 2020	https://www.addgene.org/128216/
pICH47761	Level 1 acceptor, Position 4, forward orientation	Engler et al. 2014	https://www.addgene.org/48003/
pAK-EL-03	Endlinker for tRNA-gRNA system, used for just 3 sgRNAs in the tRNA-gRNA system	Hahn et al. 2020	https://www.addgene.org/125762/
pICH47772	Level 1 acceptor, Position 5, forward orientation	Engler et al. 2014	https://www.addgene.org/48004/
pFH31	Wheat TaU3 promoter used for tRNA-gRNA system	Hahn et al. 2020	https://www.addgene.org/125884/
pICH47852	Level 1 acceptor, Position 6, reverse orientation.	Engler et al. 2014	https://www.addgene.org/48012/
pICSL12009	ZmUBI promoter + 5′UTR module	Lawrenson et al. 2015	https://www.addgene.org/68257/
pFH24	SpCas9, wheat codon optimized, version 2	Hahn et al. 2020	https://www.addgene.org/131763/
pICH41421	Nos teminator	Engler et al. 2014	https://www.addgene.org/50339/
pAGM8031	Level M acceptor, Position 6	Engler et al. 2014	https://www.addgene.org/48037/
pICH50927	Level M end-link 6	Engler et al. 2014	https://www.addgene.org/48049/
pICH47802	Level 1 acceptor, Position 1, reverse orientation.	Engler et al. 2014	https://www.addgene.org/48007/
pICH51288	2x35S promoter + 5′UTR module	Engler et al. 2014	https://www.addgene.org/50269/
TaWOX5	CDS, wheat Wuschel-related homeobox 5	Wang et al. 2022	On request
pICH41414	PolyA Terminator	Engler et al. 2014	https://www.addgene.org/50337/
pICH47742	Level 1 acceptor, Position 2, forward orientation	Engler et al. 2014	https://www.addgene.org/48001/
pICH51266	Promoter (1.3 kb), 35S (Cauliflower Mosaic Virus) + 5′UTR omega	Engler et al. 2014	https://www.addgene.org/50267/
pICSL80036	Hygromycin phophotransferase II	Lawrenson et al. 2015	https://www.addgene.org/68259/
pICH47822	Level 1 acceptor, Position 3, reverse orientation	Engler et al. 2014	https://www.addgene.org/48009/
pFH56	OsActin promoter	Hahn et al. 2020	https://www.addgene.org/125882/
pICSL80005	CDS, variant of turboGFP codon-optimised for plants	Engler et al. 2014	https://www.addgene.org/50322/

Supplementary Table S3. Primers used for detection of genome editing.

Gene	Genotype	Primer name	Primer sequences (5′–3′)	Product size (bp)	Restriction enzyme	Reference
Hpt	Fielder, Kronos	Hpt_F	AGGCTCTCGATGAGCTGATGCTTT	335	–	This study
		Hpt_R	AGCTGCATCATCGAAATTGCCGTC			This study
Qsd1	Fielder, Kronos	Qsd1-ABD_F	CAGCCTGGAGGGAATGACC	186	*Pst* I	Abe et al. 2019
		Qsd1-ABD_R	ACCTGGTGGAATCCAGAGC			Abe et al. 2019
PCL-A1	Fielder, Kronos	PCL-A1_F	ATCGGGTTGGGGATCCAATC	1065	–	This study
		PCL-A1_R	TCTTTGACTGATGGACACACACCTAC			This study
PCL-B1	Fielder	PCL-ABD_F	AAACGGGTGCGACGAGCTGACCCC	929	–	This study
		Fd_PCL-B1_R	GACACAAACACACGCCTATGCTAG			This study
PCL-B1	Kronos	Kr_PCL-B1_F	GGATTGGTGTTCATAGGTTCGGTTG	1005	–	This study
		Kr_PCL-B1_R	AACACACAGTTGTGACAGAGAAGC			This study
PCL-D1	Fielder	PCL-D1_F	TTGGGGATCCAATCCGTCCA	1058	–	This study
		PCL-D1_R	GATCGAGACACACACAGGCCTAT			Komura et al. 2024
BBX-A19	Fielder	Fd_BBX-A19_F	GTCAAGGCACAATCTTCATCTTCCAG	792	–	This study
		Fd_BBX-A19_R	GGTGGCAAATCCAGACTCATGGA			This study
BBX-A19	Kronos	BBX19-ABD_F	ATGTGTAACAAGCTTGCTAGTCGG	828	–	This study
		BBX-A19_R	TGAGATTCCAATGTTTACTTGTCGGTG			This study
BBX-B19	Fielder	BBX19-ABD_F	ATGTGTAACAAGCTTGCTAGTCGG	726	–	This study
		Fd_BBX-B19_R	AGTCTGGATTGTCGCCAGGAAG			This study
BBX-B19	Kronos	BBX19-ABD_F	ATGTGTAACAAGCTTGCTAGTCGG	720	–	This study
		Kr_BBX-B19_R	CTGGATTGTCACCAAGAGTAACGG			This study
BBX-D19	Fielder	BBX19-ABD_F	ATGTGTAACAAGCTTGCTAGTCGG	835	–	This study
		BBX-D19_R	TTTACATTCCGAGATTCCGACATTCAC			This study

Supplementary Table S4. Estimation of genome editing efficiency for each gRNA.

Genotype	Target gene	Homoeoloci	Editing efficiency[a]					
			gRNA1	gRNA2	gRNA3	gRNA4	gRNA5	gRNA6
Fielder	*PCL1*	A	100% (9/9)	87.5% (7/8)	87.5% (7/8)	75% (6/8)	71.4% (5/7)	55.6% (5/9)
		B	20% (2/10)[b]	100% (9/9)	80% (8/10)	40% (4/10)	66.7% (6/9)	80% (8/10)
		D	0% (0/11)[b]	90.9% (10/11)	62.5% (5/8)	77.8% (7/9)	90.9% (10/11)	54.6% (6/11)
	BBX19	A	73.3% (11/15)	60% (12/20)	28.6% (4/14)	35.7% (5/14)	66.7% (8/12)	30% (6/20)
		B	73.3% (11/15)	55% (11/20)	50% (9/18)	37.5% (6/16)	50% (8/16)	65% (13/20)
		D	64.3% (9/14)	0% (0/14)[c]	64.3% (9/14)	44.4% (4/9)	37.5% (3/8)	64.3% (9/14)
Kronos	*PCL1*	A	100% (14/14)	100% (14/14)	100% (8/8)	75% (6/8)	100% (12/12)	85.7% (12/14)
		B	91.7% (11/12)	100% (7/7)	100% (8/8)	77.8% (7/9)	80% (8/10)	66.7% (8/12)
	BBX19	A	81.3% (13/16)	94.1% (16/17)	81.8% (9/11)	81.8% (9/11)	80% (4/5)	88.2% (15/17)
		B	72.7% (8/11)	100% (15/15)	80% (8/10)	55.6% (5/9)	75% (3/4)	100% (15/15)

[a] Number of edited alleles / all detected alleles, except for alleles in which the target site has been deleted by other gRNAs.

[b] gRNA1 of PCL1 targets only A-homeoloci.

[c] gRNA2 of BBX19 targets A- and B-homoeoloci.

Supplementary Table S5. Comparison of the morphology of plants with and without T-DNA in ‘Fielder’ and ‘Kronos’.

Genotype	Fielder	Fielder	Kronos	Kronos
T-DNA	–	+	–	+
No. of samples	5	5	5	5
Days to heading	37.6 ± 4.12	36.8 ± 3.06	37.8 ± 0.4	37.6 ± 0.49
Spikelet number per spike	18.0 ± 2.48	15.8 ± 2.14	14.0 ± 0	11.6 ± 0.49***
Tiller number	4.6 ± 1.72	4.8 ± 1.72	4.2 ± 0.4	4.0 ± 0.63
Culm length (cm)	52.15 ± 6.01	53.22 ± 8.05	50.88 ± 5.24	53.18 ± 2.4
Culm diameter (mm)	2.33 ± 0.14	2.23 ± 0.22	1.86 ± 0.09	1.81 ± 0.1
Leaf length (cm)				
1st leaf	12.52 ± 1.08	14.56 ± 0.74**	12.66 ± 1.00	17.99 ± 1.89**
2nd leaf	21.74 ± 0.45	22.59 ± 0.63	18.94 ± 1.73	19.74 ± 1.00
3rd leaf	25.24 ± 1.67	23.52 ± 2.73	24.06 ± 1.25	20.80 ± 1.76*
4th leaf	26.74 ± 5.24	24.96 ± 5.79	25.94 ± 1.53	24.54 ± 1.39
5th leaf	28.45 ± 4.28	25.05 ± 3.21	26.30 ± 0.94	25.03 ± 1.56
6th leaf	27.38 ± 5.45	22.37 ± 3.29	25.79 ± 1.95	23.16 ± 4.14
7th leaf	–	–	22.87 ± 2.10	21.30 ± 0.64
Flag leaf	15.28 ± 5.60	13.58 ± 5.29	13.68 ± 3.38	10.58 ± 1.95
Leaf width (mm)				
1st leaf	5.12 ± 0.92	6.70 ± 0.40***	4.74 ± 0.25	5.10 ± 0.21
2nd leaf	5.08 ± 2.22	8.74 ± 1.18**	4.50 ± 0.29	6.28 ± 0.73**
3rd leaf	6.50 ± 3.82	12.28 ± 2.78*	6.02 ± 0.46	9.58 ± 0.59***
4th leaf	8.10 ± 3.77	14.00 ± 3.11*	7.92 ± 0.55	12.06 ± 0.23***
5th leaf	9.96 ± 3.54	16.80 ± 4.17*	10.06 ± 1.34	14.74 ± 1.17***
6th leaf	11.65 ± 6.72	13.77 ± 7.69	11.24 ± 1.01	16.70 ± 0.83***
7th leaf	–	–	11.00 ± 0.16	17.48 ± 1.11**
Flag leaf	11.72 ± 2.70	20.24 ± 2.90*	12.86 ± 1.26	16.56 ± 0.97**

Significant differences between lines with or without T-DNA were indicated by asterisks (Welch's t-test; $*p < 0.05$; $**p < 0.01$; $***p < 0.001$).

Supplementary Table S6. Potential off-target of designed gRNAs in this study.

gRNA	Genotype	Off-target site	Sequence (5'–3')[a]	Edited rate[b]	Forward primer (5'–3')	Reverse primer (5'–3')
PCL1 gRNA1	Fielder	*PCL-B1*	ACGTCGAGAAGCGTCCGCCC	2/5 (40%)	AAACGGGTGCGACGAGCTGACCCC	GACACAAACACACGCCTATGCTAG
PCL1 gRNA1	Fielder	*PCL-D1*	ACGTCGAGGAGCGTGCGCCC	0/5 (0%)	TTGGGGATCCAATCCGTCCA	GATCGAGACACACACAGGCCTAT
PCL1 gRNA2	Kronos	chr3B:9573759-9573778	GCTGCACAAGCGGTTCGTGG	6/6 (100%)	GGTGTCGCTTCAGCACGTTG	TGTCGCCTCAAACCATCCAGTATT
PCL1 gRNA5	Kronos	chr2A:220104608-220104627	GGAGGCGAAGATGTCGTCGG	Not determined[c]		
PCL1 gRNA6	Kronos	chr3B:9573476-9573459	--CTACCAGCAGCAGCACAG	4/6 (66.7%)	GGTGTCGCTTCAGCACGTTG	TGTCGCCTCAAACCATCCAGTATT
BBX19 gRNA 2	Fielder	*BBX-D19*	GAACAGCCATATCACAGCTC	0/11 (0%)	ATGTGTAACAAGCTTGCTAGTCGG	TTTACATTCCGAGATTCCGACATTCAC

[a] Mismatches with gRNA were shown in red

[b] Number of edited T_0 plants / Tested T_0 plants

[c] Due to the presence of sequences with high similarity within the genome

Supplementary Table S7. Summary of genome-edited sequences in *PCL1* .

<table>
<tr><th rowspan="2">Genotype</th><th rowspan="2">Homoeoloci</th><th rowspan="2">Transgenic ID[a]</th><th rowspan="2">Allele</th><th colspan="6">Mutation in sgRNA[bc]</th></tr>
<tr><th>sgRNA1</th><th>sgRNA2</th><th>sgRNA3</th><th>sgRNA4</th><th>sgRNA5</th><th>sgRNA6</th></tr>
<tr><td rowspan="30">Fielder</td><td rowspan="9">PCL-A1</td><td rowspan="2">1</td><td>1</td><td>-1 bp</td><td></td><td></td><td></td><td></td><td></td></tr>
<tr><td>2</td><td colspan="6">-408 bp</td></tr>
<tr><td rowspan="2">2</td><td>1</td><td>-1 bp</td><td>+1 bp</td><td>-3 bp</td><td>-1 bp</td><td>+1 bp</td><td>-1 bp</td></tr>
<tr><td>2</td><td>-1 bp</td><td>+1 bp</td><td>-1 bp</td><td colspan="2">-13 bp</td><td>-3 bp</td></tr>
<tr><td>3</td><td>1</td><td>-1 bp</td><td>+1 bp</td><td>-3 bp</td><td colspan="3">-225 bp</td></tr>
<tr><td rowspan="2">4</td><td>1</td><td>+1 bp</td><td>-1 bp</td><td>+1 bp</td><td>-1bp</td><td>-2 bp</td><td></td></tr>
<tr><td>2</td><td>+1 bp</td><td>+1 bp</td><td></td><td></td><td></td><td></td></tr>
<tr><td rowspan="2">5</td><td>1</td><td>-4 bp, snp1</td><td colspan="2">-62 bp</td><td>+1 bp</td><td>+1 bp</td><td></td></tr>
<tr><td>2</td><td>-1 bp</td><td colspan="2">-62 bp</td><td>-1 bp</td><td>-1 bp</td><td>-1 bp</td></tr>
<tr><td rowspan="10">PCL-B1</td><td rowspan="2">1</td><td>1</td><td></td><td colspan="2">-63 bp</td><td></td><td colspan="2">-156 bp</td></tr>
<tr><td>2</td><td>-268 bp</td><td colspan="2">163 bp invers</td><td></td><td></td><td>+20 bp</td></tr>
<tr><td rowspan="2">2</td><td>1</td><td></td><td>+1 bp</td><td>-1 bp</td><td colspan="2">-161bp</td><td></td></tr>
<tr><td>2</td><td></td><td>+1 bp</td><td>-1 bp</td><td>-5 bp</td><td colspan="2">-156 bp</td></tr>
<tr><td rowspan="2">3</td><td>1</td><td colspan="3">-317 bp</td><td></td><td>+1 bp</td><td>-4 bp</td></tr>
<tr><td>2</td><td></td><td colspan="2">-108 bp</td><td colspan="3">-254 bp</td></tr>
<tr><td rowspan="2">4</td><td>1</td><td></td><td>+1 bp</td><td></td><td></td><td></td><td></td></tr>
<tr><td>2</td><td></td><td>-1 bp</td><td></td><td></td><td></td><td>-7 bp</td></tr>
<tr><td rowspan="2">5</td><td>1</td><td></td><td colspan="2">-63 bp</td><td colspan="2">-154 bp</td><td>-2 bp</td></tr>
<tr><td>2</td><td></td><td colspan="2">-63 bp</td><td></td><td colspan="2">156 bp</td></tr>
<tr><td rowspan="11">PCL-D1</td><td rowspan="2">1</td><td>1</td><td></td><td colspan="2">-63 bp</td><td colspan="2">-21 bp</td><td>-2 bp</td></tr>
<tr><td>2</td><td></td><td colspan="4">-168 bp</td><td></td></tr>
<tr><td rowspan="2">2</td><td>1</td><td></td><td>+1 bp</td><td></td><td colspan="2">-24 bp</td><td></td></tr>
<tr><td>2</td><td></td><td colspan="3">-146 bp</td><td>+1 bp</td><td></td></tr>
<tr><td rowspan="2">3</td><td>1</td><td></td><td colspan="2">-63 bp</td><td colspan="2">-21 bp</td><td>-2 bp</td></tr>
<tr><td>2</td><td></td><td colspan="4">-168 bp</td><td>-1 bp</td></tr>
<tr><td rowspan="2">4</td><td>1</td><td></td><td></td><td></td><td></td><td></td><td></td></tr>
<tr><td>2</td><td></td><td>+1 bp</td><td></td><td>-4bp</td><td>-4bp</td><td></td></tr>
<tr><td rowspan="3">5</td><td>1</td><td></td><td>+1 bp</td><td>-1 bp</td><td>-1 bp</td><td>+1 bp</td><td>-1 bp</td></tr>
<tr><td>2</td><td></td><td>+1 bp</td><td>-1 bp</td><td>sub 2</td><td>-16 bp</td><td>-1 bp</td></tr>
<tr><td>3</td><td></td><td>+1 bp</td><td>-1 bp</td><td></td><td>+1bp, sub1</td><td>-1 bp</td></tr>
<tr><td rowspan="26">Kronos</td><td rowspan="14">PCL-A1</td><td rowspan="2">1</td><td>1</td><td>-1 bp</td><td colspan="4">-168 bp</td><td>-53 bp</td></tr>
<tr><td>2</td><td>-1 bp</td><td colspan="2">-63 bp</td><td>-1 bp</td><td>-6 bp</td><td>-2 bp</td></tr>
<tr><td rowspan="2">2</td><td>1</td><td>-2 bp</td><td colspan="4">-168 bp</td><td>-8 bp</td></tr>
<tr><td>2</td><td>-1 bp</td><td colspan="2">-63 bp</td><td>-3 bp, sub1</td><td>+1 bp</td><td>-2 bp</td></tr>
<tr><td rowspan="2">3</td><td>1</td><td>+1 bp</td><td>-59 bp</td><td colspan="3">103 bp Invers</td><td></td></tr>
<tr><td>2</td><td>-1 bp</td><td>+1bp</td><td colspan="3">-105 bp</td><td></td></tr>
<tr><td rowspan="2">4</td><td>1</td><td>-1 bp</td><td colspan="5">-319 bp</td></tr>
<tr><td>2</td><td>+1 bp</td><td colspan="3">-146 bp</td><td colspan="2">-150 bp</td></tr>
<tr><td rowspan="2">5</td><td>1</td><td>+1 bp</td><td>+1 bp</td><td>-10bp</td><td></td><td>+1 bp</td><td>-2 bp</td></tr>
<tr><td>2</td><td>+1 bp</td><td>+1 bp</td><td>-10bp</td><td>-27bp</td><td>+1 bp</td><td>-2 bp</td></tr>
<tr><td rowspan="2">6</td><td>1</td><td>-2 bp</td><td colspan="5">-318 bp</td></tr>
<tr><td>2</td><td>-1 bp</td><td>-2bp</td><td>-7 bp</td><td></td><td colspan="2">-150 bp</td></tr>
<tr><td rowspan="2">7</td><td>1</td><td>-2 bp</td><td colspan="3">-146 bp</td><td>-5 bp</td><td>-11 bp</td></tr>
<tr><td>2</td><td>-4 bp</td><td colspan="2">-62 bp</td><td>-45 bp</td><td>-4 bp</td><td>-2 bp</td></tr>
<tr><td rowspan="12">PCL-B1</td><td rowspan="2">1</td><td>1</td><td colspan="4">-406 bp</td><td>+1 bp</td><td>-5 bp</td></tr>
<tr><td>2</td><td colspan="6">-576 bp</td></tr>
<tr><td rowspan="2">2</td><td>1</td><td>-1 bp</td><td>+1 bp</td><td colspan="3">-105 bp</td><td>-2 bp</td></tr>
<tr><td>2</td><td>-1 bp</td><td colspan="2">-62 bp</td><td>-7 bp</td><td colspan="2">-150 bp</td></tr>
<tr><td>3</td><td>1</td><td></td><td colspan="2">-63 bp</td><td></td><td></td><td></td></tr>
<tr><td rowspan="2">4</td><td>1</td><td colspan="3">-322 bp</td><td>-3 bp</td><td>-18 bp</td><td>-1 bp</td></tr>
<tr><td>2</td><td colspan="4">-406 bp</td><td>-14 bp</td><td>-1 bp</td></tr>
<tr><td rowspan="2">5</td><td>1</td><td>-2 bp</td><td>+1 bp</td><td colspan="2">-84 bp</td><td>+1 bp</td><td>-4 bp</td></tr>
<tr><td>2</td><td>-2 bp</td><td colspan="2">-61 bp</td><td></td><td></td><td></td></tr>
<tr><td rowspan="2">6</td><td>1</td><td>-1 bp</td><td colspan="2">-63 bp</td><td>-8 bp</td><td>-1 bp</td><td></td></tr>
<tr><td>2</td><td colspan="6">-576 bp, 72 bp invers</td></tr>
<tr><td>7</td><td>1</td><td>-1 bp</td><td>-33 bp</td><td>-3 bp</td><td>-15 bp</td><td>-16 bp</td><td></td></tr>
</table>

[a] Transgenetic ID is correspoinding to the number on each lane in Figure 2C.

[b] Deletion, insertion, substitution, and inversion were indicated by "-", "+", "sub", and "invers", respectively.

[c] Deletion range between multiple sgRNAs was indicated by gray background.

Supplementary Table S8. Summary of genome-edited sequences in *BBX19* .

<table>
<tr><th rowspan="2">Genotype</th><th rowspan="2">Homoeoloci</th><th rowspan="2">Transgenic ID[a]</th><th rowspan="2">Allele</th><th colspan="6">Mutation in sgRNA[bc]</th></tr>
<tr><th>sgRNA1</th><th>sgRNA2</th><th>sgRNA3</th><th>sgRNA4</th><th>sgRNA5</th><th>sgRNA6</th></tr>
<tr><td rowspan="54">Fielder</td><td rowspan="20">BBX-A19</td><td>1</td><td>1</td><td></td><td></td><td></td><td></td><td></td><td></td></tr>
<tr><td rowspan="2">2</td><td>1</td><td>-2 bp</td><td colspan="2">-35 bp</td><td colspan="3">-55 bp, sub 1bp</td></tr>
<tr><td>2</td><td>-2 bp</td><td colspan="3">-172 bp</td><td colspan="2">-36 bp, sub 1bp</td></tr>
<tr><td>3</td><td>1</td><td>-11 bp</td><td>+1bp</td><td colspan="4">-190 bp</td></tr>
<tr><td rowspan="2">4</td><td>1</td><td rowspan="2">-11 bp / -16 bp</td><td colspan="2">-35bp</td><td colspan="3">-266 bp</td></tr>
<tr><td>2</td><td colspan="3">-172 bp</td><td colspan="2">-42 bp, sub 1bp</td></tr>
<tr><td rowspan="2">5</td><td>1</td><td>-5 bp</td><td colspan="5">-220 bp</td></tr>
<tr><td>2</td><td>-5 bp</td><td>-1bp</td><td colspan="4">-180 bp, sub 4bp</td></tr>
<tr><td>6</td><td>1</td><td></td><td></td><td></td><td></td><td></td><td></td></tr>
<tr><td rowspan="3">7</td><td>1</td><td></td><td>-1 bp</td><td></td><td></td><td>-1 bp</td><td>-1 bp</td></tr>
<tr><td>2</td><td></td><td></td><td></td><td colspan="2">-12 bp</td><td>-3 bp</td></tr>
<tr><td>3</td><td></td><td></td><td>-2 bp</td><td></td><td colspan="2">-26 bp</td></tr>
<tr><td rowspan="2">8</td><td>1</td><td rowspan="2">-4 bp / -7 bp</td><td colspan="5">-221 bp</td></tr>
<tr><td>2</td><td colspan="3">-172 bp</td><td>-2 bp</td><td>-3bp</td></tr>
<tr><td>9</td><td>1</td><td>-1 bp / -4 bp</td><td colspan="2">-36 bp</td><td>-6bp</td><td colspan="2">-42 bp</td></tr>
<tr><td rowspan="2">10</td><td>1</td><td rowspan="2">WT / -6 bp</td><td></td><td colspan="4">-184 bp</td></tr>
<tr><td>2</td><td></td><td></td><td></td><td>-2bp</td><td>-4bp</td></tr>
<tr><td rowspan="3">11</td><td>1</td><td></td><td></td><td></td><td colspan="2">-10 bp</td><td>+3bp</td></tr>
<tr><td>2</td><td></td><td></td><td></td><td></td><td>+1bp</td><td>+1bp</td></tr>
<tr><td>3</td><td></td><td colspan="5">-216 bp</td></tr>
<tr><td rowspan="20">BBX-B19</td><td>1</td><td>1</td><td></td><td></td><td></td><td></td><td></td><td></td></tr>
<tr><td rowspan="3">2</td><td>1</td><td rowspan="3">-8 bp / -2 bp</td><td>-11bp</td><td>-3 bp</td><td>-7 bp</td><td colspan="2">-41 bp</td></tr>
<tr><td>2</td><td>-1 bp</td><td>+ 29 bp, sub 2bp</td><td>-7 bp</td><td colspan="2">-41 bp</td></tr>
<tr><td>3</td><td></td><td colspan="2">-149bp</td><td>-5bp</td><td>-2bp</td></tr>
<tr><td rowspan="2">3</td><td>1</td><td>-5 bp</td><td colspan="5">-220 bp</td></tr>
<tr><td>2</td><td>-5 bp</td><td colspan="2">-42 bp</td><td colspan="3">-52 bp</td></tr>
<tr><td>4</td><td>1</td><td>-3 bp</td><td>-5 bp</td><td colspan="4">-180 bp, sub 3bp</td></tr>
<tr><td rowspan="2">5</td><td>1</td><td rowspan="2">-2 bp / -5 bp</td><td colspan="4">-179 bp</td><td>-3 bp</td></tr>
<tr><td>2</td><td>-1 bp</td><td>-23 bp, sub 8bp</td><td>-6 bp</td><td colspan="2">-41 bp</td></tr>
<tr><td>6</td><td>1</td><td></td><td></td><td></td><td></td><td></td><td></td></tr>
<tr><td rowspan="2">7</td><td>1</td><td rowspan="2">WT / -5 bp</td><td></td><td></td><td></td><td></td><td></td></tr>
<tr><td>2</td><td></td><td></td><td></td><td>-2bp</td><td></td></tr>
<tr><td rowspan="2">8</td><td>1</td><td>+1bp</td><td>+1bp</td><td colspan="4">-190 bp</td></tr>
<tr><td>2</td><td>+1bp</td><td colspan="2">-36 bp</td><td></td><td>-1 bp</td><td>-3 bp</td></tr>
<tr><td>9</td><td>1</td><td>-1 bp / -4 bp</td><td>-1bp</td><td colspan="2">-140 bp</td><td colspan="2">-42 bp</td></tr>
<tr><td>10</td><td>1</td><td></td><td></td><td></td><td></td><td></td><td></td></tr>
<tr><td rowspan="4">11</td><td>1</td><td></td><td></td><td></td><td></td><td></td><td></td></tr>
<tr><td>2</td><td></td><td></td><td></td><td></td><td></td><td>-1 bp</td></tr>
<tr><td>3</td><td></td><td>-2bp</td><td></td><td></td><td></td><td></td></tr>
<tr><td>4</td><td></td><td></td><td></td><td></td><td></td><td>-2bp</td></tr>
<tr><td rowspan="14">BBX-D19</td><td>1</td><td>1</td><td></td><td></td><td></td><td></td><td></td><td></td></tr>
<tr><td rowspan="2">2</td><td>1</td><td rowspan="2">-1 bp / -5 bp</td><td></td><td colspan="4">-180 bp, sub 4bp</td></tr>
<tr><td>2</td><td></td><td colspan="3">sub 2 bp, -140 bp</td><td>+1bp</td></tr>
<tr><td rowspan="2">3</td><td>1</td><td rowspan="2">-2 bp / -6 bp</td><td></td><td>-1bp</td><td></td><td></td><td></td></tr>
<tr><td>2</td><td></td><td colspan="4">-186bp</td></tr>
<tr><td rowspan="2">4</td><td>1</td><td rowspan="2">-3 bp / +7 bp</td><td></td><td>-1bp</td><td colspan="3">-52bp</td></tr>
<tr><td>2</td><td></td><td colspan="4">-186 bp</td></tr>
<tr><td>5</td><td>1</td><td>-7 bp</td><td></td><td colspan="4">-180 bp, sub3 bp</td></tr>
<tr><td>6</td><td>1</td><td></td><td></td><td></td><td></td><td></td><td></td></tr>
<tr><td>7</td><td>1</td><td></td><td></td><td></td><td></td><td></td><td></td></tr>
<tr><td>8</td><td>1</td><td>-1 bp</td><td></td><td>-5 bp</td><td>-4 bp</td><td colspan="2">-42 bp</td></tr>
<tr><td>9</td><td>1</td><td>-1 bp</td><td></td><td>-3 bp</td><td colspan="3">-53 bp</td></tr>
<tr><td>10</td><td>1</td><td></td><td></td><td></td><td></td><td></td><td></td></tr>
<tr><td>11</td><td>1</td><td></td><td></td><td></td><td></td><td></td><td></td></tr>
</table>

Supplementary Table S8. (continued)

<table>
<tr><th rowspan="2">Genotype</th><th rowspan="2">Homoeoloci</th><th rowspan="2">Transgenic ID[a]</th><th rowspan="2">Allele</th><th colspan="6">Mutation in sgRNA[bc]</th></tr>
<tr><th>sgRNA1</th><th>sgRNA2</th><th>sgRNA3</th><th>sgRNA4</th><th>sgRNA5</th><th>sgRNA6</th></tr>
<tr><td rowspan="32">Kronos</td><td rowspan="17">BBX-A19</td><td>1</td><td>1</td><td>-3 bp / -2 bp</td><td>-2bp</td><td></td><td colspan="3">-52 bp</td></tr>
<tr><td rowspan="2">2</td><td>1</td><td rowspan="2">-4 bp / -3 bp</td><td colspan="5">sub 5bp, -231 bp</td></tr>
<tr><td>2</td><td colspan="3">-172 bp</td><td colspan="2">-41 bp</td></tr>
<tr><td rowspan="2">3</td><td>1</td><td rowspan="2">-4 bp / -5 bp</td><td>+1 bp</td><td>-1 bp</td><td colspan="3">-45 bp, sub 5bp</td></tr>
<tr><td>2</td><td colspan="5">-220 bp</td></tr>
<tr><td rowspan="2">4</td><td>1</td><td></td><td></td><td></td><td></td><td></td><td></td></tr>
<tr><td>2</td><td></td><td>sub 1bp, -33 bp</td><td>-2bp</td><td></td><td colspan="2">sub 4bp, -55 bp</td></tr>
<tr><td rowspan="3">5</td><td>1</td><td rowspan="3">WT / -21 bp</td><td colspan="2">-36 bp</td><td colspan="3">-49 bp</td></tr>
<tr><td>2</td><td colspan="2">-36 bp</td><td colspan="3">-52 bp</td></tr>
<tr><td>3</td><td colspan="2">-49 bp</td><td colspan="3">-49 bp</td></tr>
<tr><td rowspan="2">6</td><td>1</td><td rowspan="2">-2 bp / -1 bp</td><td colspan="5">-219 bp</td></tr>
<tr><td>2</td><td colspan="5">-218 bp</td></tr>
<tr><td rowspan="2">7</td><td>1</td><td rowspan="2">6 bp, sub 8bp / -4</td><td>-1 bp</td><td>-4 bp</td><td colspan="3">-53 bp</td></tr>
<tr><td>2</td><td colspan="5">-221 bp</td></tr>
<tr><td rowspan="2">8</td><td>1</td><td rowspan="2">WT / -5 bp</td><td>-18 bp</td><td colspan="2">174bp Invers</td><td colspan="2">-32 bp, sub 3bp</td></tr>
<tr><td>2</td><td>-1 bp</td><td colspan="4">-224 bp</td></tr>
<tr><td>9</td><td>1</td><td>-5 bp</td><td>-1 bp</td><td>-1 bp</td><td>-2 bp</td><td>-41 bp</td><td></td></tr>
<tr><td rowspan="15">BBX-B19</td><td>1</td><td>1</td><td>-1 bp</td><td colspan="5">-231 bp</td></tr>
<tr><td>2</td><td>1</td><td>-1 bp</td><td colspan="5">43 bp invers, -154 bp</td></tr>
<tr><td rowspan="2">3</td><td>1</td><td>-22 bp</td><td>-1 bp</td><td>-5 bp</td><td colspan="3">-51 bp</td></tr>
<tr><td>2</td><td>-22 bp</td><td colspan="2">-36 bp</td><td colspan="3">-47 bp</td></tr>
<tr><td rowspan="3">4</td><td>1</td><td></td><td>+1 bp</td><td></td><td></td><td></td><td>-1 bp</td></tr>
<tr><td>2</td><td></td><td>-1 bp</td><td></td><td></td><td>-1 bp</td><td>-3 bp</td></tr>
<tr><td>3</td><td></td><td>-1 bp</td><td>-1 bp</td><td></td><td>-1 bp</td><td>-3 bp</td></tr>
<tr><td>5</td><td>1</td><td>-28 bp, sub 1bp</td><td>-2 bp</td><td colspan="4">-186 bp</td></tr>
<tr><td>6</td><td>1</td><td>WT /-1 bp</td><td colspan="2">-35 bp</td><td colspan="3">-47 bp</td></tr>
<tr><td rowspan="2">7</td><td>1</td><td>-1 bp</td><td>-1 bp</td><td>-4 bp</td><td colspan="3">-51 bp</td></tr>
<tr><td>2</td><td>-1 bp</td><td>sub 1bp, -1 bp</td><td>-2 bp</td><td colspan="3">-55 bp</td></tr>
<tr><td rowspan="2">8</td><td>1</td><td></td><td colspan="5">-220 bp</td></tr>
<tr><td>2</td><td></td><td colspan="2">-35 bp</td><td></td><td colspan="2">sub 3bp, -43 bp</td></tr>
<tr><td rowspan="2">9</td><td>1</td><td rowspan="2">-7 bp / -122 bp</td><td colspan="5">-221 bp</td></tr>
<tr><td>2</td><td colspan="5">-320 bp</td></tr>
</table>

[a] Transgenetic ID is correspoinding to the number on each lane in Figure 2C.

[b] Deletion, insertion, substitution, and inversion were indicated by "-", "+", "sub", and "invers", respectively.

[c] Deletion range between multiple sgRNAs was indicated by gray background.

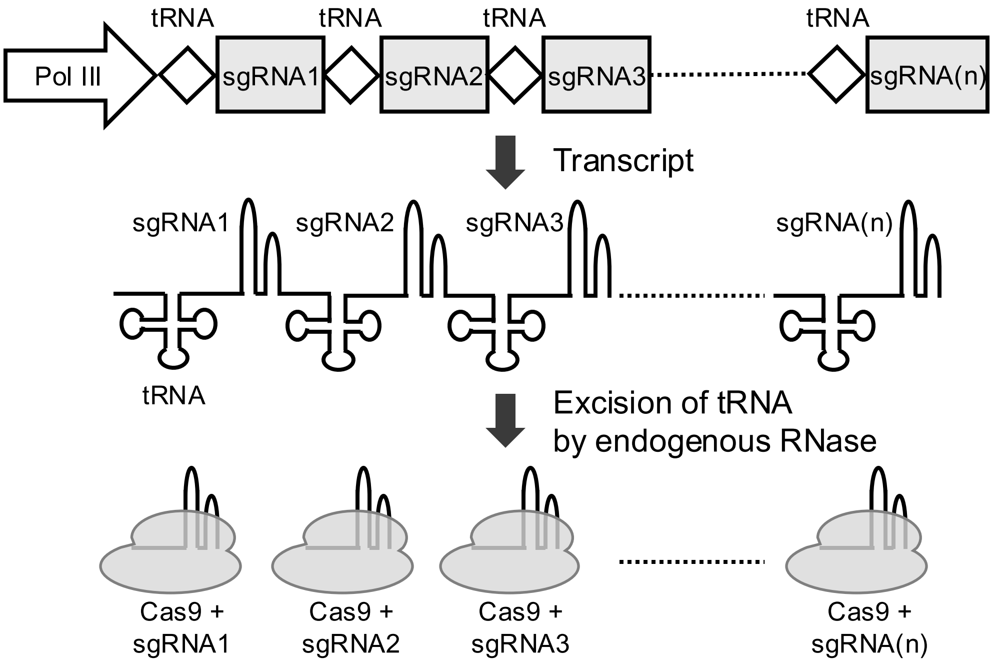

Supplementary Figure S1. Overview of the endogenous tRNA processing system of CRISPR/Cas9 genome editing (Xie et al. 2015).

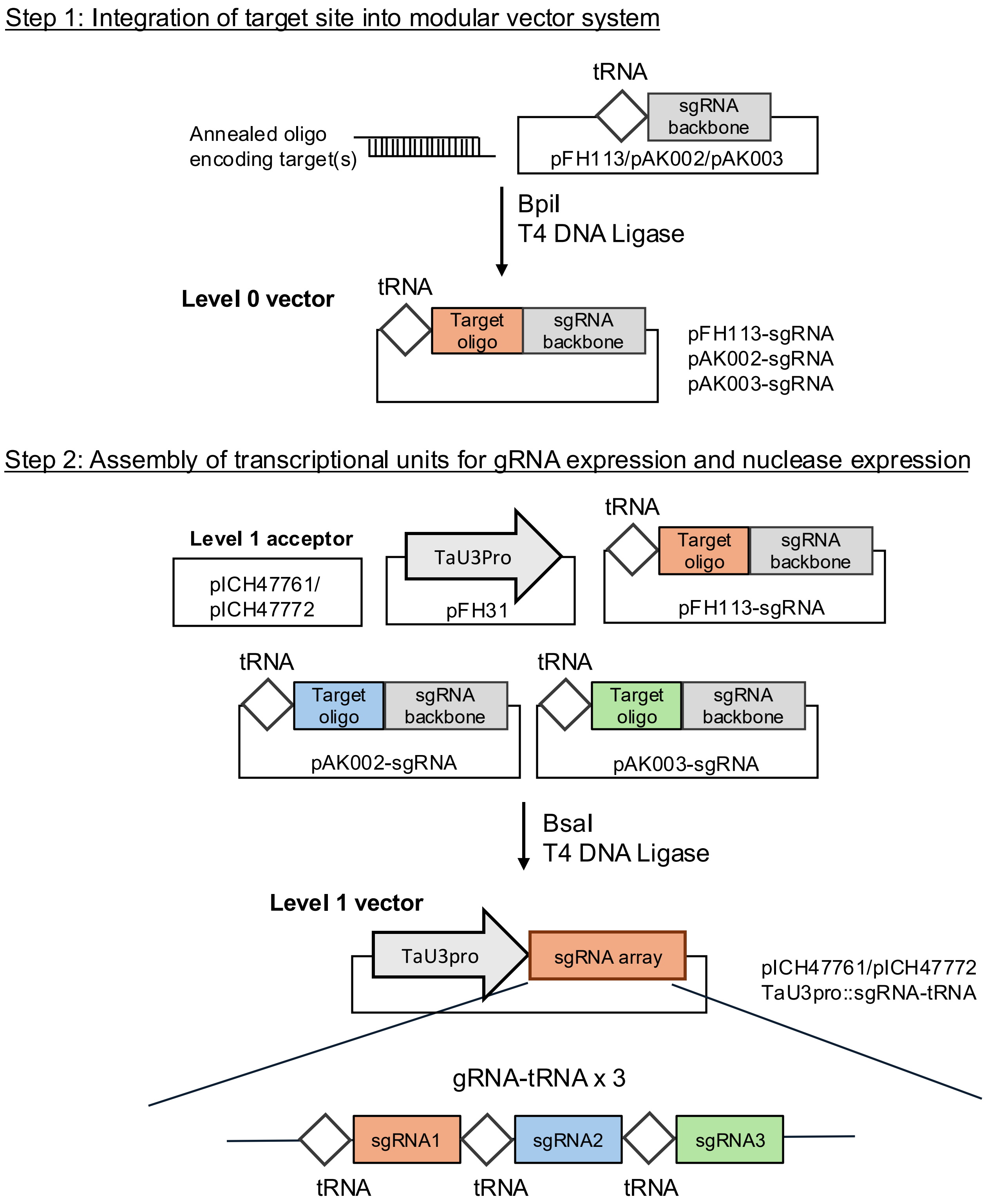

Supplementary Figure S2. Schematic representation of vector assembly for tRNA–gRNA expression system based on the Golden Gate cloning method (Engler et al., 2014; Hahn et al., 2020). 35Spro, CaMV 35S promoter; *Hpt*, hygromycin phosphotransferase II; NosT: nopaline synthase terminator; OsActinPro, *Oryza sativa* actin promoter; *SpCas9*, *Streptococcus pyogenes* Cas9; T-35S, CaMV35S terminator; TaU3pro, TaU3 promoter; tRNA, transfer RNA; UbiPro, ubiquitin promoter from *Zea mays*; *TaWOX5*, WUS homeobox-containing 5 from *Triticum aestivum*.

Step 3: Assembly of transcriptional units

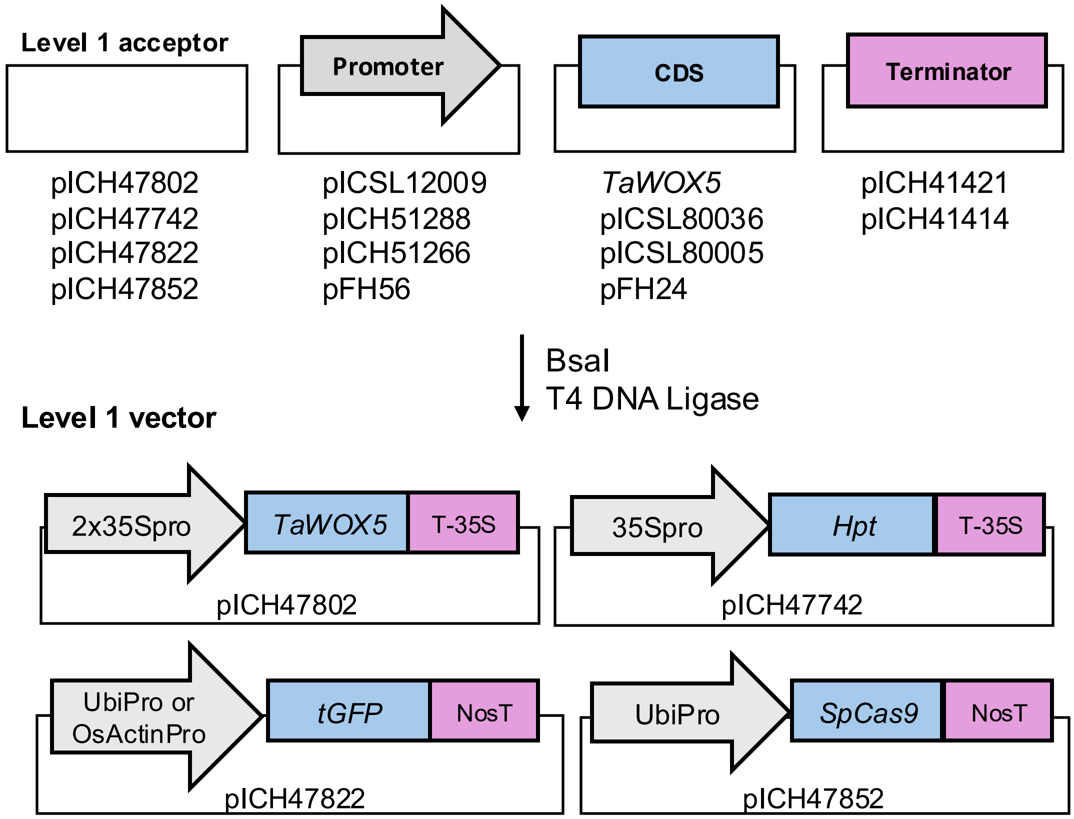

Supplementary Figure S2. *Continued.*

Step 4: Assembly of multi gene construct

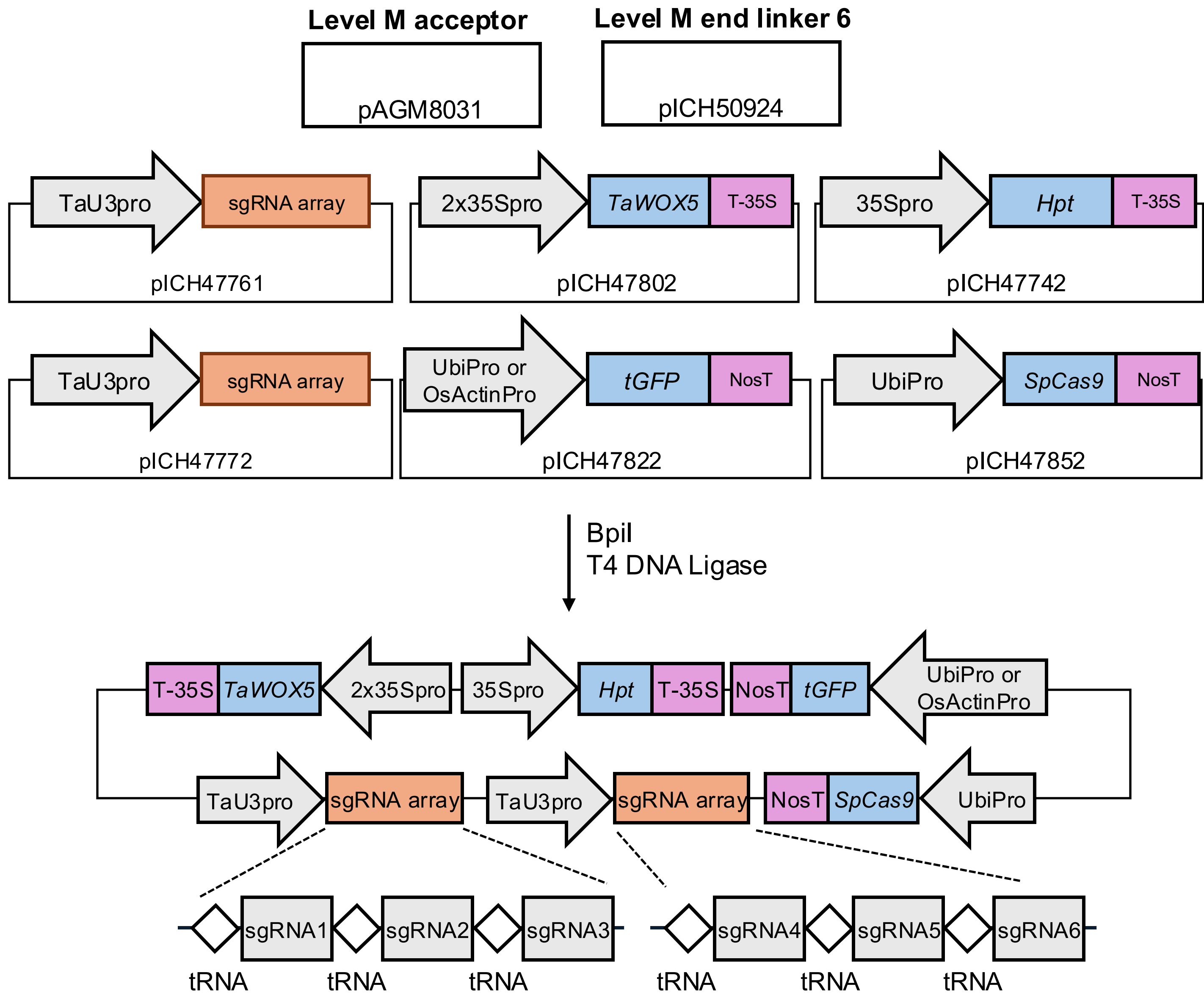

Supplementary Figure S2. *Continued.*

Qsd-A1

```
WT      CCTTCCCACGGATCCACCTCCCTGCAGCGGCGATCAAAGCCGCCAAGGC
pos1-1  CCTTCCCACGGATCCACCTCCCTGACAGCGGCGATCAAAGCCGCCAAGG  (+1bp)
pos1-3  CCTTCCCACGGATCCACCTCCCT-CAGCGGCGATCAAAGCCGCCAAGGC  (-1bp)
pos2-2  CCTTCCCACGGATCCACCTCCC--CAGCGGCGATCAAAGCCGCCAAGGC  (-2bp)
        CCTTCCCACGGATCCACCTCCCTG--------------GCCGCCAAGGC  (-14bp)
        CCTTCCCA-------------------CGGCGATCAAAGCCGCCAAGGC  (-19bp)
pos2-4  CCTTCCCACGGATCCACCTCC---CAGCGGCGATCAAAGCCGCCAAGGC  (-3bp)
        CCTTCCCACGGATCCACC------CAGCGGCGATCAAAGCCGCCAAGGC  (-6bp)
pos3-2  CCTTCCCACGGATCCACCTCC----AGCGGCGATCAAAGCCGCCAAGGC  (-4bp)
pos3-5  CCTTCCCACGGATCCACCTCCCTGCAGCGGCGATCAAAGCCGCCAAGGC  (WT)
        CCTTCCCACGGATCCACCTCCCT-CAGCGGCGATCAAAGCCGCCAAGGC  (-1bp)
```

Qsd-B1

```
WT      CCTTCCCACGGATCCACCTCCCTGCAGCGGCGATCAAAGCCGCCAAGGC
pos1-1  CCTTCCCACGGATCCACCTCCCT-CAGCGGCGATCAAAGCCGCCAAGGC  (-1bp)
        CCTTCCCACGGATCCACCTCC---CAGCGGCGATCAAAGCCGCCAAGGC  (-3bp)
pos1-3  CCTTCCCACGGATCCACCTCCCT-CAGCGGCGATCAAAGCCGCCAAGGC  (-1bp)
pos2-2  CCTTCCCACGGATCCACCTCCCTGCAGCGGCGATCAAAGCCGCCAAGGC  (WT)
        CCTTCCCACGGATCCACCTCCCTGCCAGCGGCGATCAAAGCCGCCAAGGC  (+1bp)
pos2-4  CCTTCCCACGGATCCACCTCC----AGCGGCGATCAAAGCCGCCAAGGC  (-4bp)
pos3-2  CCTTCCCACGGATCCACCTCCCTGGCAGCGGCGATCAAAGCCGCCAAGGC  (+1bp)
pos3-5  CCTTCCCACGGATCCACCTCCC---AGCGGCGATCAAAGCCGCCAAGGC  (-3bp)
```

Qsd-D1

```
WT      CCTTCCCACGGATCCACCTCCCTGCAGCGGCGATCAAAGCCGCCAAGGC
pos1-1  CCTTCCCACGGATCCACCTCC---CAGCGGCGATCAAAGCCGCCAAGGC  (-3bp)
        CCTTCCCACGGATCCACCTCCCTGCCAGCGGCGATCAAAGCCGCCAAGGC  (+1bp)
pos1-3  CCTTCCCACGGATCCACCTCCCTGACAGCGGCGATCAAAGCCGCCAAGGC  (+1bp)
pos2-2  CCTTCCCACGGAT--------------CGGCGATCAAAGCCGCCAAGGC  (-14bp)
        CCTTCCCACGGATCCACCTCCCTGCCAGCGGCGATCAAAGCCGCCAAGGC  (+1bp)
pos2-4  CCTTCCCACGGATCCACCTC------GCGGCGATCAAAGCCGCCAAGGC  (-6bp)
pos3-2  CCTTCCCACGGATCCACCTCCCT-CAGCGGCGATCAAAGCCGCCAAGGC  (-1bp)
        CCTTCCCACGGATCCACCTCCCTGCCAGCGGCGATCAAAGCCGCCAAGGC  (+1bp)
pos3-5  CCTTCCCACGGATCCACCTCCCTGTCAGCGGCGATCAAAGCCGCCAAGGC  (+1bp)
```

Supplementary Figure S3. Mutations detected in T_1 plants derived from selected T_0 plants transformed with the three sgRNA modules (pos1–3). Sequence names correspond to the transgenic IDs depicted in Figure 1C.

References in supplementary materials

Abe F, Haque E, Hisano H, Tanaka T, Kamiya Y, Mikami M, Kawaura K, Endo M, Onishi K, Hayashi T, Sato K (2019) Genome-Edited Triple-Recessive Mutation Alters Seed Dormancy in Wheat. Cell Rep 28:1362-1369.e4

Engler C, Youles M, Gruetzner R, Ehnert TM, Werner S, Jones JDG, Patron NJ, Marillonnet S (2014) A Golden Gate modular cloning toolbox for plants. ACS Synth Biol 3:839–843

Hahn F, Korolev A, Loures LS, Nekrasov V (2020) A modular cloning toolkit for genome editing in plants. BMC Plant Biol 20, 179

Komura S, Yoshida K, Jinno H, Oono Y, Handa H, Takumi S, Kobayashi F (2024) Identification of the causal mutation in early heading mutant of bread wheat (*Triticum aestivum* L.) using MutMap approach. Mol Breed 44, 41

Lawrenson T, Shorinola O, Stacey N, Li C, Østergaard L, Patron N, Uauy C, Harwood W (2015) Induction of targeted, heritable mutations in barley and Brassica oleracea using RNA-guided Cas9 nuclease. Genome Biol 16, 258

Wang K, Shi L, Liang X, Zhao P, Wang W, Liu J, Chang Y, Hiei Y, Yanagihara C, Du L, Ishida Y, Ye X (2022) The gene *TaWOX5* overcomes genotype dependency in wheat genetic transformation. Nat Plants 8:110–117

Xie K, Minkenberg B, Yang Y (2015) Boosting CRISPR/Cas9 multiplex editing capability with the endogenous tRNA-processing system. Proc Natl Acad Sci U S A 112:3570–3575
